# Standardization and quality management in next-generation sequencing

**DOI:** 10.1016/j.atg.2016.06.001

**Published:** 2016-07-01

**Authors:** Christoph Endrullat, Jörn Glökler, Philipp Franke, Marcus Frohme

**Affiliations:** Molecular Biotechnology and Functional Genomics, Institute of Applied Biosciences, Technical University of Applied Sciences Wildau, Hochschulring 1, 15745 Wildau, Germany

**Keywords:** ABRF, Association of Biomolecular Resource Facilities, BAM, **b**inary **a**lignment/**m**ap, CAP, College of American Pathologist's, CEN, European Committee for Standardization, CLIA, Clinical Laboratory Improvement Amendments, ddPCR, digital droplet PCR, ERCC, External RNA Controls Consortium, FDA, Food and Drug Administration, FFPE, formalin-fixed, paraffin-embedded, FMEA, **f**ailure **m**ode and **e**ffects **a**nalysis, GATK, **g**enome **a**nalysis **t**oolkit, GSC, Genomic Standards Consortium, HGP, Human Genome Project, Indel, insertion or deletion, MAQC, MicroArray Quality Control Project, MIGS, **m**inimum **i**nformation about a **g**enome **s**equence, MOL, molecular pathology checklist, mtDNA, mitochondrial DNA, Nex-StoCT, **n**ext **g**eneration **s**equencing — **s**tandardization of **c**linical **t**esting, NGS, next-generation sequencing, NIST, National Institute of Standards and Technology, NTC, no-template control, PT, **p**roficiency **t**esting, QA, quality assurance, QC, quality control, QM, quality management, QMS, quality management system, qPCR, quantitative PCR, RIN, RNA **i**ntegrity **n**umber, SAM, **s**equence **a**lignment/**m**ap, SEQC, **s**equencing **q**uality **c**ontrol, SNP, single nucleotide polymorphism, SOP, standard operating procedure, TN, technical note, VCF, **v**ariant **c**all **f**ormat, Next-generation sequencing, Quality management, Data quality, Standardization, Validation, Guideline

## Abstract

DNA sequencing continues to evolve quickly even after > 30 years. Many new platforms suddenly appeared and former established systems have vanished in almost the same manner. Since establishment of next-generation sequencing devices, this progress gains momentum due to the continually growing demand for higher throughput, lower costs and better quality of data. In consequence of this rapid development, standardized procedures and data formats as well as comprehensive quality management considerations are still scarce. Here, we listed and summarized current standardization efforts and quality management initiatives from companies, organizations and societies in form of published studies and ongoing projects. These comprise on the one hand quality documentation issues like technical notes, accreditation checklists and guidelines for validation of sequencing workflows. On the other hand, general standard proposals and quality metrics are developed and applied to the sequencing workflow steps with the main focus on upstream processes. Finally, certain standard developments for downstream pipeline data handling, processing and storage are discussed in brief. These standardization approaches represent a first basis for continuing work in order to prospectively implement next-generation sequencing in important areas such as clinical diagnostics, where reliable results and fast processing is crucial. Additionally, these efforts will exert a decisive influence on traceability and reproducibility of sequence data.

## 1 Introduction

**Fig. 1:**
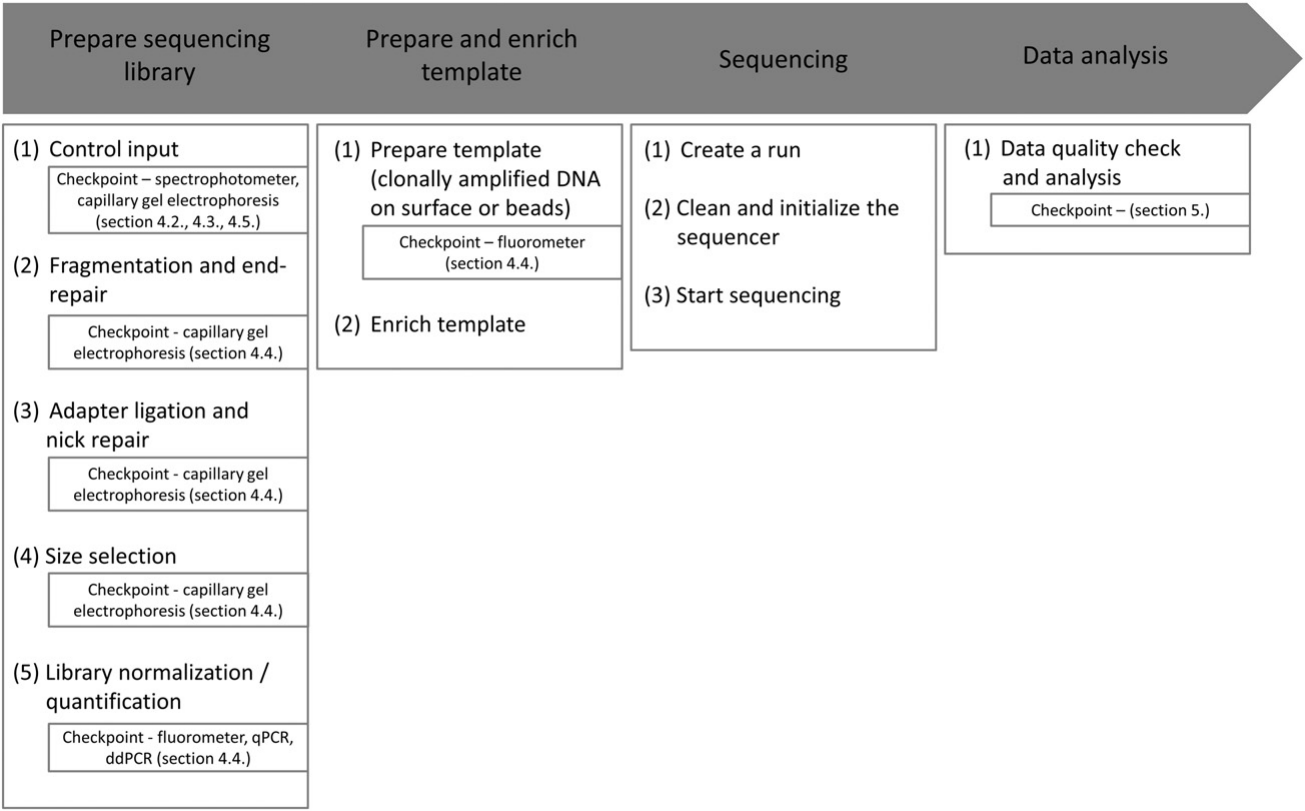
Overview of the general NGS workflow. The main steps library and template preparation, enrichment, sequencing and data analysis are divided into substeps containing recommendations for checkpoints which are proposed for QC.

The initial sequencing methods were developed by Maxam and Gilbert as well as Sanger and Coulson with the latter being almost the only method in use for > 30 years (Hutchison, 2007, Schuster, 2008). These methods became popular, because they represented the first approaches for unraveling DNA nucleotide sequences. Since 2005, second-generation sequencing technologies, termed as next-generation sequencing (NGS), allow to investigate whole genomes or transcriptomes from different organisms in relatively short time (Morozova and Marra, 2008, Pareek et al., 2011). The promise to get an insight into gene function and regulation led to an increasing number of methods and systems in the last ten years. These new methods and systems steadily increased in throughput, broad in field of applications and resulted in better quality of data (Metzker, 2010). Consequently, a big market has established comprising sequencing services providers and sequencing platform manufacturers. By implication, NGS exerts nowadays an increasing impact on academic research, diagnostics and industry. In particular, the establishment of NGS in diagnostics will entail many advantages such as higher throughput of patient samples and thus obtaining faster results and decreased costs (Dietel et al., 2015, Meldrum et al., 2011), because today's medical questions are focusing on therapy opportunities for complex genetic diseases (Boycott et al., 2013, ten Bosch and Grody, 2008). Especially in oncology, the perspective of finding a universal agent in form of a single pharmaceutical drug is obsolete and there is an increasing demand for new innovations regarding appropriate therapeutic treatments in order to promote the upcoming field of personalized medicine (Ross and Cronin, 2011). Unfortunately, the current high costs of NGS and uncertainty of data quality (O'Rawe et al., 2015) precludes the unfettered use in diagnostics at this stage and appropriate projects are neither still not certified by the US Food and Drug Administration (FDA) (Gargis et al., 2012) nor regulated under the Clinical Laboratory Improvement Amendments (CLIA) guidelines (Westgard and Westgard, 2006). Moreover, the implementation of guidelines often collides with individual demands and issues of single laboratories as well as organizations and many research units have little or any experience with quality management (QM) and quality assurance (QA). In order to change this, the standardization and simplification of NGS workflows is a central requirement, involving QM and QA methods. Standards act as basic guidelines to ensure comparability and exchange of experimental data conducive to accelerate the innovation process and aid improvement of transferability, transparency and reproducibility of results. Furthermore, standardization potentially realizes a higher turnover by decreasing costs, therefore enabling an improved financial planning and scheduling and thus a possible expansion of services in an industrial context. However, standardization is a complex topic characterized by several problems and challenges like failure of initializing developed standards, missing consensus and deadlocks as well as incompatible implementations of finished standards (Cargill, 2011). In particular, the formulation of NGS standards requires additionally an extensive collection and evaluation of appropriate platform-dependent and independent information as well as comparative analysis of different sequencing systems. To comply with aforementioned points, our aim was to identify previously established standards, recommendations and guidelines for workflows, generally addressing genomic DNA sequencing, and the corresponding QM and QA opportunities, which are summarized within the framework of this article. The results gained from successful standardization of NGS may transfer to other fields in life sciences. An overview of the general NGS workflow annotated with single steps and appropriate QC checkpoints is given in Fig. 1.

## 2 Quality management (QM) and quality assurance (QA) in NGS

A good starting point in standardization measures is the introduction of quality documentation. Frequently, there is an obvious lack of such documentation within sequencing experiments. Thus, it is crucial to develop and establish procedure-, operating- and inspection instructions as well as quality records. Furthermore, verification documents, particularly for providing a string of documents for the verifiable origin of sequencing data is an essential point. Especially the quality records could act as a certificate for customers and the general documentation would improve the traceability and transparency with the aim to prove the reliability of results. Another important parameter in QM considerations is the QA. A QA program should contain predetermined quality control (QC) checkpoints for monitoring QA and an extensive documentation including, among others, used devices, reagent lot numbers and any deviation from standard procedures (Gargis et al., 2012, Rehm et al., 2013). Moreover, the QA program should contain QC methods for contamination identification at several stages within the sequencing workflow. These stages comprise the initial sample evaluation, the fragmentation step, the final library assessment, the monitoring of error rates during the sequencing process and the raw data analysis with focus on read quality (Rehm et al., 2013).

### 2.1 The technical note (TN) as a QA method to fulfill QM documentation

In order to address the aspects, mentioned in Section 2, the establishment of a TN is recommended. The TN acts as a preventive QA method, respectively a guideline to avoid common problems- and error-sources like the performance of single preparation steps in different laboratories or companies, and to remedy the lack of documentation. It is conceivable as a docket or inspection record, which is permanently carried along in order to ensure comprehensive QM documentation. After completion of a sequencing project, the TN represents additionally a quality certificate for delivery to the customer. A generic TN for the sample fragmentation step is shown in the Appendix (see Appendix A — Table A.1).

### 2.2 Laboratory accreditation checklist requirements by the College of American Pathologist's (CAP) NGS work group

The CAP NGS Work Group also works on means of quality documentation, but in a broader context, overarching general QA. They developed 18 laboratory accreditation checklist requirements for upstream analytic processes and downstream bioinformatics solutions for NGS in clinical applications (Aziz et al., 2015). These requirements were published within CAP's molecular pathology checklist (MOL) and include new standards for documentation, validation, QA, confirmatory testing, exception logs, monitoring of upgrades, variant interpretation and reporting, incidental findings, data storage, version traceability and data transfer confidentiality. The wet bench process comprise workflow steps such as handling of patient samples, extraction of nucleic acids, fragmentation, bar coding, optional enrichment of targets, adaptor ligation, amplification, library preparation, flow cell loading and generation of sequence reads (Aziz et al., 2015). The MOL topics for the wet bench process are summarized in the Appendix (see Appendix A — Table A.2).

### 2.3 Guidelines for validation of sequencing workflows in clinical applications

The promising establishment of NGS into clinical practice led to a wealth of considerations regarding the formulation of strict guidelines and regulations by different bodies (Bennett and Farah, 2014). One of the first legislated guidelines are the “NGS guidelines for somatic genetic variant detection”, provided by the New York State Department of Health (http://www.wadsworth.org/sites/default/files/WebDoc/1300145166/NextGenSeq_ONCO_Guidelines.pdf). These guidelines include validation requirements and usage of reference materials. The former covers key performance indicators such as accuracy (recommended minimum of 50 samples composed of different material type), robustness (likelihood of assay success), precision (recommended minimum of three positive samples for each variant type), repeatability and reproducibility (ability to return identical results under identical or changed conditions) as well as analytical sensitivity and specificity (positive and negative percent of results compared to gold standard). Accuracy, sensitivity and specificity in NGS assays are based on depth of coverage and quantity of reads associated with a respective base call (Gargis et al., 2012). Other validation parameters like repeatability and reproducibility, which are required elements for establishing precision in NGS tests, must be determined by sequencing the same reference sequence several times under same conditions (repeatability) respectively under changed conditions (reproducibility), i.e. processing the upstream pipeline in multiple laboratories while utilizing different devices (Gargis et al., 2012). Together with both reportable and reference range the aforementioned validation requirements represent additionally the performance characteristics as published in the CLIA guidelines (Westgard and Westgard, 2006). The clinical laboratory demands are divided into QA, validation, data, QC and reports, while the quality management system (QMS) is characterized by a three tier hierarchy including policies, standard operating procedures (SOP) and records. However, the main focus of QM systems for clinical NGS applications relies on SOP's. In addition, the proposed criteria for platform selection are total sequence capacity, sequence read length, sequence run time and the final quality and accuracy. These criteria are also essential for performance optimization approaches. While the QA serves as establishment of quality infrastructure, the QC is valuable to confirm testing outputs against requirements. Finally, the recommended QC reference materials are no-template controls (NTC), which have to be embedded into all amplifications steps, the negative control for initial and periodically validation and the positive/sensitivity control, which must be determined for each sequencing run (https://www.horizondiscovery.com/reference-standards/what-are-reference-standards/quality-controlled/new-york-state-guidelines). A similar approach was performed by Gargis et al. who developed principles, guidelines, standards as well as recommendations for the implementation of NGS into diagnostic laboratories within the Nex-StoCT project (Next Generation Sequencing - Standardization of Clinical Testing) (Gargis et al., 2012). A major obstacle in NGS standardization represents the absence of an established Proficiency Testing (PT) system, which causes lack of error identification, missing indication of QC problems as well as aggravated verification of test performance in laboratories. Therefore, the Nex-StoCT workgroup developed and published recommendations for the structure of a novel NGS PT program. These recommendations comprise on the one hand the establishment of a methods-based assessment for test performance in order to improve inter-laboratory comparisons by using exclusively genomic DNA from well-characterized cell lines as PT samples. On the other hand, they suggested the utilization of electronic data as PT samples to evaluate the downstream bioinformatics abilities of different users. Hence, the Nex-StoCT group proposed PT opportunities for both wet and dry laboratory pipelines thus covering the entire NGS workflow (Gargis et al., 2012).

## 3 Standardization efforts from organizations and companies

Next to the aforementioned efforts there are other standardization approaches, especially from public institutes and societies. The US National Institute of Standards and Technology (NIST) focused on standardization of sample preparation within the framework of very diverse projects. The most promising ongoing project is the “Genome in a Bottle Consortium” (https://sites.stanford.edu/abms/giab). This consortium centers its attention on adapting procedures established for whole genome sequencing to the clinical environment by investigation of reference data, methods and standards for NGS. Another standardization approach is running by the Association of Biomolecular Resource Facilities (ABRF), a network between different research departments which address several biomolecular issues regarding standardization and optimization with the objective to develop guidelines. Especially the ABRF-NGS group has to be taken into account, due to their work on identification of optimal methods and strategies for NGS projects as well as performance evaluation of different NGS platforms. The main study of this work group is divided into two phases involving RNA sequencing with focus on utilizing reference samples such as standardized genomic DNA and synthetic spike-in RNA controls (Li et al., 2014, Tighe et al., 2013). The basis of the aforementioned ABRF study represents the MicroArray Quality Control Project (MAQC), which addressed the reliability and reproducibility of cross-platform gene expression analysis as well as development of standards and quality guidelines (MAQC Consortium et al., 2006). Especially the third phase of MAQC has to be considered, which is called Sequencing Quality Control (SEQC/MAQC-III) (SEQC/MAQC-III Consortium, 2014). SEQC/MAQC-III centers on evaluation of technical performance between different NGS platforms by establishing benchmarks with reference samples.

## 4 Standard proposals for general sequencing workflows

### 4.1 Composition of NGS workflows

All NGS workflows can be divided into pre-analytical, analytical and post-analytical process steps, where different standards are applicable. While pre-analytical standards aim at quality, format and amount of specimen/sample, which should be documented in detail, the analytical standards consider the proof of these aspects, concerning DNA/RNA extraction, quantification and purity determination via fluorometry or spectrophotometry. If a sample does not meet the defined minimum requirements, the first most recommended way is to refuse processing of the sample, respectively order a new one (Rehm et al., 2013). Therefore, Pacific Biosciences defined five specific questions regarding the overall sample characterization in order to ensure appropriate QC procedures (http://jgi.doe.gov/wp-content/uploads/2013/11/Importance-of-Sample-QC.pdf). Firstly, they require knowing the original source of the sample (blood, tissue, etc.). Secondly, they request which methods were used to isolate the sample and thirdly, which quantification was performed (Qubit or NanoDrop). The last both points address questions such as, if there was a quality assessment via gel electrophoresis and whether a clean-up procedure took place before shearing.

### 4.2 Standard proposals for sample preparation step

Ensuring a good DNA/RNA quality begins already during isolation and extraction. It is required to keep the majority of incubation steps at lower temperatures (< 60 °C), inhibit or buffer possible nuclease activity while storing DNA/RNA samples permanently on ice and avoid repeated freeze-thawed cycles. (http://www.mscience.com.au/upload/pages/pacbio/technical-note---experimental-design-for-microbial-assembly-2012.pdf). Furthermore, it has to be taken into account, that there are some special sample quality requisitions for third-generation sequencing systems like Pacific Biosciences RS II, owing to omission of DNA amplification. These requisitions comprise, among others, double-stranded format of DNA, prevention of pH extremes (< 6/> 9), absence of chelating agents, detergents, divalent metal cations, denaturants or RNA, respectively carryover contaminants from starting material (http://www.umich.edu/~caparray/products/ngs/pacbio/Pacific%20Biosciences%20Template%20Preparation%20and%20Sequencing.pdf). The quality assessment should yield an OD_260/280_ ratio of 1.8 to 2.0 and an OD_260/230_ ratio of 2.0 to 2.2 with latter being an additional value for purity determination. Moreover, it is recommended to perform an initial DNA damage repair for genomic DNA sequencing applications and the quality of DNA should be always assessed prior library preparation (i.e. via capillary gel electrophoresis) (http://www.pacb.com/wp-content/uploads/2014/04/TemplatePreparation.pdf). This makes it obvious that the DNA input amount as well as following amplification steps are major bias-related factors. Dependent on application it is recommend using 30–50 ng of DNA input and omission of PCR to avoid e.g. GC bias during the library preparation (Chen et al., 2014).

### 4.3 Quality evaluation of formalin-fixed, paraffin-embedded (FFPE) RNA samples

Commonly used is the RNA Integrity Number (RIN) for the quality evaluation of RNA calculated by the 28S peak area divided by the 18S peak area and an undisclosed variable (Schroeder et al., 2006). As an alternative the DV_200_ is a reliable QC value especially for quantification of FFPE RNA samples, which is calculated straightforward via e.g. Agilent Bioanalyzer or Advanced Analytical Fragment Analyzer and involves the complete electropherogram above fragment sizes of 200 bases (= smear analysis) (Wang et al., 2016). On the one hand, a high percentage of fragments > 200 nucleotides represents a high RNA integrity and thus a better quality. On the other hand, a low percentage relates to higher degradation and lower quality. The DV_200_ shows a considerably higher reliability in comparison to RIN regarding RNA quality determination and thus allows the preparation of valuable libraries out of poor source material (Eikrem et al., 2016).

### 4.4 Standard devices for sample quality assessment

There are two different devices often stated in NGS vendor manuals respectively sequencing protocols, frequently found in nearly every laboratory and thus almost exclusively used for QC in sequencing projects. The first is the capillary gel electrophoresis, which is embedded in the overwhelming majority of projects and studies for investigation of fragment size distribution as well as final library quality assessment (Borgström et al., 2011). Therefore, such device like Agilent Bioanalyzer can be considered as an unofficial standard for QC and the obtained results should be deposited in order to fulfill good QM documentation. To determine the input DNA/RNA amount and to check sample quality at appropriate steps of the sequencing workflow, the second most commonly used appliance represents the fluorometer, which offers fluorometric quantitation. Most frequently used devices for this purpose are the Thermo Fisher Scientific Qubit Fluorometer and the NanoDrop. Hence, there are two different options available for quality assessment during defined sequencing workflow steps, nonetheless it is recommend utilizing both appliances (Simbolo et al., 2013). Since most sequencing protocols recommend at least one of both devices for QC, it is up to the respective laboratory whether they use only one or both, dependent on their quality standards and regulations. Moreover, for accurate quantification of DNA/RNA at certain workflow steps and for determining the final library quantity, established systems such as quantitative PCR (qPCR) or digital droplet PCR (ddPCR) are recommended (Robin et al., 2016).

### 4.5 Spike-in controls for downstream quality evaluation

Another recommended and already established standard is the spike-in control. It is a matter of a well-known and validated reference DNA sample which ensures the quality evaluation at the end of a sequencing workflow in order to identify errors during data analysis (Ledergerber and Dessimoz, 2011). This control is carried along the whole process and undergoes the same handling steps as the investigated sample, from initial quantification to final downstream processing. If a sequence error is observed in the reference control, the same error occurred in all likelihood in the main sample. Therefore, the spike-in control is considered to be a benchmark for sample quality. A suitable reference is the genomic DNA of bacteriophage φX174. Due to commensurable straightforward cultivation and the quite small genome of merely 5386 nucleotides (Michel et al., 2010), which needs much less space on a sequencing flow cell, the use of φX174 is convenient. Moreover, RNA sequencing applications offer already a set of established RNA spike-in controls developed by the External RNA Controls Consortium (ERCC) (Baker et al., 2005). These RNA standards consist on the same principles as the above mentioned DNA controls but undergo in contrast more handling steps of library preparation and deliver therefore a better performance reflection of the endogenous sample (Jiang et al., 2011).

## 5 Standard proposals for sequence data handling, processing and storage

### 5.1 The impact and classification of sequencing errors

With establishment of NGS some new challenges were ahead. In comparison to largely standardized Sanger sequencing, the quality per base was generally lower, which decreased the specificity of polymorphism detection. Sanger sequencing is considered as being a well-established automated sequencing method and as current gold standard for variant identification and it is possible to get access to well characterized reference samples where reliable data are available in order to ensure analytical validity (Grada and Weinbrecht, 2013, McCourt et al., 2013, Tsiatis et al., 2010). Due to novel high-throughput opportunities, the pooling of samples was in many cases required to improve the efficiency but led on the other hand to different concentrations between different samples. This influenced the sensitivity of a given assay. Additionally, biases and miscalls, respectively undercalls and overcalls occurred during target enrichment via PCR because of polymerase errors (Brockman et al., 2008). Compared to Sanger sequencing, polymerase errors in NGS have an impact on the overall error rate of the system. One of the main error sources represents the noise in a system, which is produced through different aspects within a run. In case of 454 platforms these aspects includes optical and chemical noise, multiple templates on one bead, signal contamination from nearby wells and a loss of synchrony between the large amount of template copies on each bead as well as homopolymeric sequence runs (Brockman et al., 2008). Moreover, Schmutz et al. proposed different error definitions and error events in a bioinformatics context (Schmutz et al., 2004). Contiguous insertion, deletion or an erroneous run of multiple base pairs were defined as a single error event, whereas a misassembly considered whole sequences. A significant error counts as a single error as well but was defined as at least 50 contiguous incorrect base pairs, which led to the definition of the base pair error.

### 5.2 Downstream bioinformatics pipeline and data analysis

The advent of NGS technologies led to a lot of different file formats, some of them established as standards or *de facto* standards over time. One of these common file formats represents FASTQ. Each sequencing platform is able to generate a FASTQ file during the downstream processing, which is equipped with the Phred score, an associated per base quality score which is based on an estimated error probability (Cock et al., 2010) (see Appendix A — Table A.3). The FASTQ file format was established for functioning as an extension to FASTA, characterized by aforementioned Phred score for each base in a sequence, plus an optional line for comments. This optional comment/description line should be thereby standardized by containing all additional and essential information regarding the sample. Currently, the FASTQ format and corresponding Phred scores are not determined as official standards, but actually work as unwritten *de facto* standards for base qualities due to most widely acceptance as cross-platform interchange file format, since establishment of Sanger sequencing (Cock et al., 2010). Moreover, QUAL is another introduced file format, which stores appropriate Phred scores and accompanies to FASTA files, especially in NGS. Access to particular Phred quality scores might be beneficial for objective comparisons between different sequencing platforms and represents directly a criterion for QC, respectively quality documentation. The equivalent *de facto* standard for variant calls is the Variant Call Format (VCF), which established during the 1000 Genomes Project and plays a pivotal role especially in clinical sequencing applications (Rehm et al., 2013). A VCF file stores information about sequence variations like Indels (insertions or deletions) or single nucleotide polymorphisms (SNP's) together with comprehensive annotation (Danecek et al., 2011). An additional standardized file format which emerged during the 1000 Genomes Project, represents the Sequence Alignment/Map (SAM) format, which includes read alignments against a reference sequence, whereby SAM is nowadays substituted, respectively used in addition to the Binary Alignment/Map (BAM) format, the compressed analogue to the SAM format (Li et al., 2009). The generated output files after a sequencing run have to be analyzed and annotated in the downstream bioinformatics pipeline using appropriate software. One common tool is FASTQC, which evaluates the quality of sequencing results for FASTQ files using statistical tests (http://www.bioinformatics.babraham.ac.uk/projects/fastqc/). However, there is a vast variety of additional QC software available, such as NGS QC Toolkit (Patel and Jain, 2012), QC-Chain (Zhou et al., 2013) or ChromaPipe (Otto et al., 2008). For specific VCF data validation and annotation, regarding variant calls, multiple realignments or genotyping, the Genome Analysis Toolkit (GATK) represents an often used framework (McKenna et al., 2010).

### 5.3 Data submission requirements and standards

After completion of data QC, analysis and annotation, the next step faces the submission of final files to customers or public databases. To address the standard minimum requirements for submission of generated and downstream analyzed, respectively annotated NGS data, Chain et al. recommend an overall coverage of at least 90% for sequence data and a form of gap resolution to minimize the number of contigs and scaffolds (Chain et al., 2009). Additionally, they suggest the verification and correction of annotation procedures regarding anomalies in coding regions to improve the comparability of genes. To address especially the genomic sequence annotation, the Genomic Standards Consortium (GSC) developed and published the Minimum Information about a Genome Sequence (MIGS) specification in order to remedy the lack of incomplete genome descriptions (Field et al., 2008). Due to the emerging field of metagenomics, adequate sequence descriptions are crucial for respective approaches. Besides common sequencing parameters like depth of coverage or overall quality, the MIGS specification also lists information referring to a broader biological context such as taxonomy, trophic level or propagation. Moreover, the current gold standard for sequence data is described with properties such as at most 1 error per 10,000 base pairs (pursuant to 99.99% accuracy) and assembly of each replicon into a single contig, while all sequences are complete and have been reviewed and edited (Chain et al., 2009, Schmutz et al., 2004). The determined accuracy of 99.99% is part of the Bermuda Standards, which were established during the Human Genome Project (HGP) meeting in 1997 and acts as a standard for sequence fidelity (http://web.ornl.gov/sci/techresources/Human_Genome/research/bermuda.shtml). The second Bermuda Standard prescribes that the sequence should be contiguous, so gaps are not left out. Consensus accuracy, contiguity and fidelity are thus gold standards which were defined in a relatively early state of genome sequencing (Schmutz et al., 2004).

### 5.4 Further considerations for NGS data handling

Standardization of genome source, library construction, hierarchical sequencing strategies and definition of what means “finished”, paired with a centralized QC center are additional suggestions with the aim to improve sequence quality (Schmutz et al., 2004). Especially the centralized QC center would exert an advantageous influence on sequence fidelity by evaluating different techniques, rather than independent technique examination by each center for itself. Consequently, this center could distribute reviews and test performance reports for technological developments in order to serve each prospect sequencing service with up to date information and innovations. Furthermore, the coverage across the sample and the percentage of bases that meet the required minimum coverage threshold are among those aspects, which should define a high quality value for different samples. Therefore, every laboratory is encouraged to set a minimum coverage, especially in medical applications, where high quality variant calls are an absolute requirement. Additionally, the percentage of aligned reads, percentage of unique reads, percentage of bases corresponding to targeted sequences, uniformity of coverage, density of clusters and percentage of targeted bases with no coverage are possible data quality features (Rehm et al., 2013).

## 6 Conclusions

There is still a long road ahead to the establishment of a general standard in NGS. The first problem faces the standardization contributions from a global point of view. NGS originated from the US and shows the broadest distribution there, thus the overwhelming majority of standardization efforts are based overseas. In contrast, verifiable respectively published approaches from other countries do not exist at this time. Therefore, international initiatives should be found and encouraged to participate in this field, especially addressing the considerable presence of European standardization bodies like the European Committee for Standardization (CEN). An additional obstacle of standardization represents the validity of standards across different NGS applications. It became obvious that the current focus relies on NGS standardization in clinical diagnostics due to highest demands and requirements regarding QC and data reliability in this area. The same standards which will be determined for clinical sequencing would not be necessarily applicable or reasonable in e.g. plant genome sequencing and vice versa. However, once standardization reaches an advanced status the established standards will be adopted for other applications, whereby the formulation of standards will likely accelerate at this stage. In order to address opportunities for future work in NGS standardization, one reasonable next step could be the development of a comprehensive Failure Mode and Effects Analysis (FMEA) in contemplation of standardizing QA aspects for NGS. The FMEA serves as a fault prevention strategy for recognition of potential error sources and the immediate reaction to these errors at an earliest stage within a process. The authors developed a first draft of a FMEA dealing with standardization of QA aspects for ion semiconductor sequencing, which is on request available. Moreover, once standardization reaches a more sophisticated level, the subsequent step will be the automation of whole sample and library preparation on a consolidated platform. This will introduce the feasibility to parallelize several platform-independent NGS workflows conducive to improving cost and time efficiency as well as increasing throughput. Finally, in order to mention an upcoming application of NGS, Parson et al. performed a project to evaluate high-throughput mitochondrial DNA (mtDNA) sequencing useful for forensic analysis (Parson et al., 2013). Originally managed by Sanger sequencing the nowadays possibilities of NGS enable expeditiously and economical investigation of mitochondrial genome information as it is already achieved by Illumina's recent MiSeq FGx sequencer (Caratti et al., 2015). These specific targets provide haplotype-specific patterns of mutations and thus build the basis for QC of novel mtDNA data in order to apply NGS in forensics (Parson et al., 2013).

## Figures and Tables

**Table A.1:** Questions and specifications of a generic TN for the fragmentation step in NGS experiments. The official approval number is assembled out of sample strain notation and date of order, whereby precise sample identification is possible at each process step. The last row comprises only the bare necessities with reference to other appropriate TN's (i.e. TN with detailed specifications and parameters about a device or kit). Another important aspect concerns the documentation of barcode sequences within the TN in order to track pooled samples (Head et al., 2014).

Question	Specification
Who performed fragmentation of the sample?	1) Name, title 2) Position 3) Areas of responsibility
What sample was fragmented?	1) Sample source 2) Starting material 3) Official approval number
When was the sample fragmented?	1) Time 2) Date 3) Signature
Where was the sample fragmented?	1) Company 2) Department 3) Laboratory 4) Postal address
Why was the sample fragmented?	1) Order number 2) Date of order
How was the sample fragmented?	1) Devices/Materials/Kits 2) Device parameters 3) Results of quantification 4) Specification of quality controls 5) References

**Table A.2:** Upstream analytical process accreditation requirements as published by CAP's NGS Work Group MOL (Aziz et al., 2015). The table summarizes seven laboratory checklist requirements for the wet bench process in NGS experiments.

MOL topic	Description	Requirements
Documentation	Use of SOP	All standard operating protocols must be documented in order to trace each step and manipulations All used methods, reagents, instruments, instrument software and versions have to be documented Controls need to be described Targeted NGS assays and target-enrichment protocols, regarding captured regions must be documented Development of SOP for each validated sample type Sequence information of barcodes for pooled analysis must be documented Metrics and QC parameters regarding run performance have to be documented (% of reads mapping to target region, base quality and coverage thresholds, average coverage/base and target region) Definition and documentation of acceptance and rejection criteria for sample preparation and sequencing Documentation of regions that failed analysis Written SOP for evidence of compliance
Validation	Validation and revalidation of processes after establishment of modifications	Analytic performance of NGS procedures must be validated Determination of optimal assay conditions and analysis settings in order to optimize each workflow step Determination of performance parameters (sensitivity, specificity, accuracy, reproducibility, reliability, detection limit) Validation of each sample type Determination of a minimum number of samples, dependent on sample, target, assay and application Revalidation of minor changes such as new reagent charges Documentation of lot numbers Revalidation of major changes such as new sequencing system or different target enrichment method
QM	Use of documented QM program	Development of a QM plan QA program must be written and compliance has to be documented QA program should assess all parts of a workflow QA program should be embedded within overall QMS Quality program should address general problems, especially in case of nonconformance with laboratories own policies and procedures Quality program should encourage scientific and laboratory staff to interchange about quality issues
Confirmatory testing	Use of policy for documentation of confirmatory testing	Established policy that clearly documents indications for confirmatory testing Each laboratory has to show evidence of monitoring their NGS assays
Laboratory records	Use of laboratory records for identification and trace of samples	Documentation of all reagents, primers, sequencing chemistries and platforms Description of performed test comprising targeted sequence and depth of coverage Citing publications or websites Maintaining a documentation system
Exception log	Use of exception log for monitoring deviations from SOP	Documentation of any deviation from SOP, the reason for deviation and the outcome Extensive consultation with laboratory supervisor or laboratory director regarding testing issues Documentation of troubleshooting, resolution and appropriate communications Changes or modifications in SOP should be supported by two qualified staff members and have to be signed by the laboratory director
Monitoring of upgrades	Use of policy for monitoring, implementing and documentation of upgrades	Implementation of policy to monitor and integrate upgrades to instruments, sequencing chemistries and reagents/kits Validation of introduced up-to-date instruments or reagents/kits Addressing methods which are used to monitor upgrades

**Table A.3:** Overview of Phred quality scores and the corresponding base calling accuracies. The table shows different Phred scores, the probability of an incorrect base call as well as the respective accuracy per base for the appropriate quality scores. Q30, i.e. 99.9% accuracy or 1 error per 1000 bases, is generally considered to be a benchmark for quality (http://www.illumina.com/documents/products/technotes/technote_Q-Scores.pdf).

Phred quality score	Probability of incorrect base call	Base call accuracy
10	1 in 10	90%
20	1 in 100	99%
30	1 in 1000	99.9%
40	1 in 10,000	99.99%
50	1 in 100,000	99.999%
